# Colorectal Cancer Detection via Metabolites and Machine Learning

**DOI:** 10.3390/cimb46050254

**Published:** 2024-04-30

**Authors:** Rachel Yang, Igor F. Tsigelny, Santosh Kesari, Valentina L. Kouznetsova

**Affiliations:** 1 REHS Program, San Diego Supercomputer Center, University of California San Diego, MC 0505, 9500 Gilman Drive, La Jolla, CA 92093, USA; 2 San Diego Supercomputer Center, University of California San Diego, MC 0505, 9500 Gilman Drive, La Jolla, CA 92093, USA; vkouznetsova@ucsd.edu; 3 BiAna, P.O. Box 2525, La Jolla, CA 92038, USA; 4 Department of Neurosciences, University of California San Diego, MC00505, 9500 Gilman Drive, La Jolla, CA 92093, USA; 5 CureScience Institute, 5820 Oberlin Drive, STE 202, San Diego, CA 92121, USA; 6 Pacific Neuroscience Institute, 2125 Arizona Avenue, Santa Monica, CA 90404, USA; santosh.kesari@providence.org

**Keywords:** colorectal cancer, data mining, machine learning, metabolites, pathway analysis

## Abstract

Today, colorectal cancer (CRC) diagnosis is performed using colonoscopy, which is the current, most effective screening method. However, colonoscopy poses risks of harm to the patient and is an invasive process. Recent research has proven metabolomics as a potential, non-invasive detection method, which can use identified biomarkers to detect potential cancer in a patient’s body. The aim of this study is to develop a machine-learning (ML) model based on chemical descriptors that will recognize CRC-associated metabolites. We selected a set of metabolites found as the biomarkers of CRC, confirmed that they participate in cancer-related pathways, and used them for training a machine-learning model for the diagnostics of CRC. Using a set of selective metabolites and random compounds, we developed a range of ML models. The best performing ML model trained on Stage 0–2 CRC metabolite data predicted a metabolite class with 89.55% accuracy. The best performing ML model trained on Stage 3–4 CRC metabolite data predicted a metabolite class with 95.21% accuracy. Lastly, the best-performing ML model trained on Stage 0–4 CRC metabolite data predicted a metabolite class with 93.04% accuracy. These models were then tested on independent datasets, including random and unrelated-disease metabolites. In addition, six pathways related to these CRC metabolites were also distinguished: aminoacyl-tRNA biosynthesis; glyoxylate and dicarboxylate metabolism; glycine, serine, and threonine metabolism; phenylalanine, tyrosine, and tryptophan biosynthesis; arginine biosynthesis; and alanine, aspartate, and glutamate metabolism. Thus, in this research study, we created machine-learning models based on metabolite-related descriptors that may be helpful in developing a non-invasive diagnosis method for CRC.

## 1. Introduction

Colorectal cancer (CRC) is the second leading death-causing cancer for men and women in the United States combined. It is a cancer originating in the colon or rectum, where the cells start to grow out of control. Colon cancer most prominently affects older people, 55 years and up. Death rates have been improving for decades due to the enhanced accuracy in detection and increased participation in recommended screening tests for adults 45 years and older. Still, the American Cancer Society estimates that in 2023, colorectal cancer will cause around 52,550 deaths [1]. Ultimately, this number can be further reduced with continued advancements in the efficacy of screening methods, as early detection has the highest success rate at saving lives by quickly treating possibly cancerous polyps before the cancer can spread further.

At present, the most common and sensitive screening test in use is colonoscopy. This method utilizes a long, flexible tube—the scope—attached with a camera, allowing doctors to view the entire colon and rectum and to remove polyps and abnormal tissue samples, at the same time, during a screening session. However, colonoscopy has risks and drawbacks, such as being invasive, requiring a diet change, its use of sedation, internal bleeding, tearing of the colon or rectum, etc. Thus, in recent research, a new, highly potential way to identify this cancer earlier has emerged—metabolomics, especially, analyzing metabolites—as a non-invasive detection approach.

Metabolomics in the field of cancer research can be used to identify the existence of cancerous cells through closely analyzing the behavior of metabolites—substances that result from metabolism—in the body. Patient samples are taken, and studies track values such as fold change/induction, *p*-values, etc. By comparing metabolites of healthy patients to cancer patients, researchers can distinguish metabolites that indicate the presence of cancerous cells or irregularities in metabolism. With more studies performed, the list of known metabolites that are most significantly and directly involved in colorectal cancer will increase, and thus metabolite analysis is a promising choice for future colorectal cancer diagnosis.

In a study, 886 plasma metabolites were identified through mass spectroscopy; for analysis, a statistical model based on conditional logistic regression was used to approximate odds ratios adjusted for multiple variables. They reached 95% confidence in predicting the risk of CRC for each increase in one standard deviation, albeit finding that six metabolites were deemed connected to CRC risk at a false discovery rate (FDR) of less than 0.20 [2]. A sample from 110 patients and control serums showed that using ^1^H-NMR to analyze the metabolites proved promising for early detection. Twenty-three metabolites were distinguished through multivariate statistical analysis (MSA), including revealing that pyruvate and glycerolipid metabolisms are activated in polyps, while in CRC, the serine, glycine, glycolysis, and threonine metabolisms were found to be activated [3]. A follow-up replication study identified differential metabolites using statistical tests (variable importance of projection score, VIP > 1 and *p* < 0.05) to demonstrate that serum biomarkers are promising for diagnosing CRC non-invasively [4]. Overall, previous metabolomics studies have demonstrated considerable success and indicated new directions for further research.

AI, and especially machine learning, is currently widely used for diagnostics. One of the useful methods for the extraction and interpretation of images ready to computer-classification is radiomics, which can be used as a necessary step of preprocessing data for machine learning [5].

## 2. Materials and Methods

### 2.1. Approach Overview

**Figure 1:**
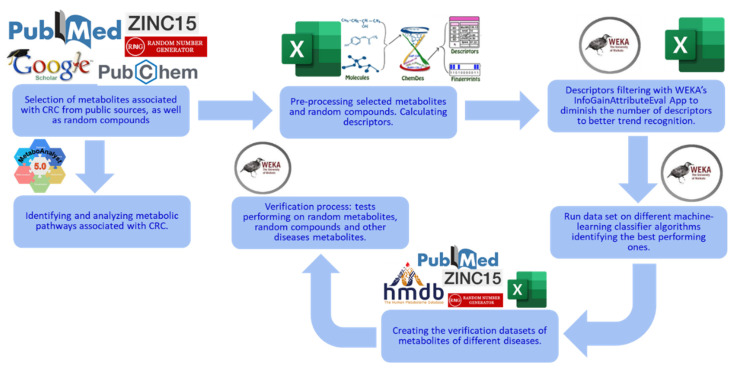
Overview of this study’s methods. This study starts with data collection: CRC-related metabolites are obtained and used as the selected metabolites [13]. Random compounds are obtained from ZINC as the control group. Descriptors are then calculated from ChemDes and filtered using the InfoGainAttributeEval application in WEKA. ML models are built and tested with multiple classification algorithms and a 10-fold cross-validation strategy in WEKA. In MetaboAnalyst, metabolic pathways related to the CRC metabolites are found, analyzed, and then visualized. Finally, the best-performing machine-learning models are tested on new independent data, including random and unrelated-disease metabolites.

The metabolite datasets used in this study were extracted from public sources and two databases, the Human Metabolome Database (HMDB), version 5.0 [6,7] and ZINC-22 [8]. The software used for machine learning was as follows: Waikato Environment for Knowledge Analysis (WEKA), version 4.2 [9], ChemDes, version 1.5 [10], and MetaboAnalyst, version 5.0 for the analysis of metabolic pathways [11,12]. The flowchart of the methods used in this study is shown in Figure 1.

### 2.2. Metabolite Selection

We began this study with the selection of metabolites associated with CRC from serum samples [13]. The details of metabolite extraction and GC/MS methods are described in the articles referenced in this source [13]. This dataset contained metabolites categorized into three groups: Stages 0–2, which include individuals without cancer and patients with Stage 1 and 2 CRC; Stages 3–4, which include patients with Stage 3 and 4 CRC; and Stages 0–4, which include the whole cohort. This dataset also contained their fold change and *p*-values. Metabolites with *p*-values *p* ≥ 0.05 were considered insignificant and filtered out. The remaining metabolites were used for ML model development. The resulting dataset contained 67 CRC metabolites in the Stage 0–2 category, 73 CRC metabolites in the Stage 3–4 category, and 79 CRC metabolites in the Stage 0–4 category. These metabolites were labeled as “selected” for ML model development.

To create the control group, randomly selected compounds were extracted from the ZINC database. The selected metabolites are presented in the Supplemental Materials (Tables S1–S3).

Then, using ChemDes, 3679 descriptors were calculated for each metabolite and compound, which were filtered using the InfoGainAttributeEval application in WEKA, leaving 937 significant descriptors.

For the three datasets—Stages 0–2, Stages 3–4, and Stages 0–4—different classification methods were tested on each to discover which method yielded the highest accuracy, using WEKA’s classify tool.

To test the best-performing ML models on unseen, independent data, we compiled three additional test sets: a set containing 79 random metabolites obtained from HMDB, another containing 79 random compounds from ZINC, and a final one containing 23 metabolites associated with thyroid cancer (to show selectivity for CRC cancer) [14].

### 2.3. ChemDes

ChemDes is a web-based platform that calculates molecular descriptors and fingerprints [10]. ChemDes was used to calculate 1D and 2D descriptors of the training set metabolites from the SMILES [15,16], which were retrieved from PubChem 2023 release [17].

### 2.4. Human Metabolome Database (HMDB)

HMDB is a database that stores information on human body-related metabolites [6,7]. We used the HMDB to create a random metabolite dataset for ML model development.

### 2.5. MetaboAnalyst

MetaboAnalyst 5.0 [11,12] is a program for statistical, functional, and integrative analysis of metabolomics data. It has four main functions of exploratory statistical analysis, functional enrichment analysis, data integration and systems biology (biomarker analysis, pathway analysis, and network explorer), and data processing. The program takes various types of input data, including compound names, KEGG ID, release 110.0, [18,19], or Human Metabolite Database index (HMDB ID) numbers [6,7] to support integrative analysis with transcriptomics or metagenomics.

### 2.6. PubChem

PubChem is a database that provides accessibility to information on an expansive selection of chemicals [17]. For this study, we utilized PubChem’s feature of readily stored simplified molecular-input line-entry system (SMILES) values for each compound [15,16]. The SMILES values of most selected and random metabolites were obtained this way.

### 2.7. Drift

The Drift software tool, 2.3.9, is used to predict protein targets for small molecules [20]. The program creates a two-dimensional fingerprint (FP2) with more than 1000 features for the selected compound. Such fingerprints were created for the following main databases: ChEMBL, v, 24, [21], ZINC [8], HMDB [6,7] and BindingDB, release 2023, [22,23]. Similarity of a fingerprint of the analyzed compound to any of these db compounds fingerprints is calculated with the Tanimoto coefficient. A sequence convolution and graph convolution neural networks are used for predicting the binding score of a compound with a protein.

### 2.8. PANTHER

The PANTHER software, version 18.0, contains a knowledge base about protein families and their evolutionary history. These phylogenetic trees are annotated by GO annotations. Panther enrichment analysis is one of the tools that can help in the analysis of genes datasets [24].

### 2.9. STRING

STRING, version 12.0, is a network generating program based on the information of protein–protein physical and functional interactions collected from various sources [25]. The program uses automated scientific texts mining, co-expression-based predictions of interactions, interaction experimental data, signaling pathways information, and more. When one uses metabolites or other entities as biomarkers, it always conjures the following question: how are these biomarkers related to the studied process? STRING outlined the clusters of genes interacting with the elected metabolites, showing their involvement in cancer-related pathways.

### 2.10. DisGeNET

DisGeNET, version 7.0, is a software application that elucidates information on genes and their variants with human diseases [26]. It is based on a collection of gene/disease and variant/disease data extracted from scientific literature using text mining. It uses a set of deep learning subsystems to generate the final information.

### 2.11. Machine-Learning Analysis (WEKA)

Machine-learning (ML) model development was performed with the Waikato Environment for Knowledge Analysis (WEKA) software [9]. WEKA is a workbench that supports, among others, multiple ML classification algorithms. The short descriptions of used ML classifiers are presented in Table S4. It contains tools for data pre-processing, classification, regression, clustering, association rules, and visualization. One of the important algorithms in WEKA is InfoGainAttributeEval. This algorithm is used for attribute (descriptor) selection, which was performed using the ranker search method. The training dataset includes both selected metabolites and random compounds. It was run on various classifiers to find the best-performing model. We tested several classification methods and selected the methods giving higher accuracy. Finally, the best model was tested on a dataset of random and unrelated-disease metabolite sets to test the final accuracy. To achieve the most effective supervised machine learning possible, we needed to prepare as much-detailed training data patterns as possible. Any selection of “average” values of the data can lead to the loss of information. During the process of learning, the ML system usually discards descriptors that not significantly impact the classification process.

### 2.12. PathBIX

The PathBIX program, release 2021, performs pathway annotation using network-based tools along with FunCoup networks, KEGG, Reactome, and WikiPathways databases [27].

### 2.13. Method Limitations

A limitation to our method may be that we included an initial list of metabolites that was too short; This list was used for training the descriptors patterns. It can artificially diminish the presence of specific descriptors that are important for general recognition.

## 3. Results

The final dataset used to construct the ML models contained 67 selected metabolites and 67 random compounds for Stages 0–2, 73 selected metabolites and 73 random compounds for Stages 3–4, and 79 selected metabolites and 79 random compounds for Stages 0–4 after filtering out selected metabolites with p-values greater than 0.05.

Using the MetaboAnalyst pathway analysis tool, we studied the metabolic pathways related to CRC in each of the groups of stages.

### 3.1. Metabolic Pathways Related to CRC Metabolites

We performed pathway analysis on the datasets of CRC-related metabolites used for the ML models’ construction. The goal was to show that these compounds could serve as solid biomarkers of CRC and that they play a significant role in cancer development. The following pathways were found to be related to these sets of metabolites.

#### 3.1.1. Aminoacyl-tRNA Biosynthesis

A study by Zhou et al. [28] explains importantance of Aminoacyl-tRNA in protein synthesis, with regard to how RNA genetic information is transferred into amino acids, and may play a role in tumorigenesis or that formation of cancer, including CRC.

#### 3.1.2. Glyoxylate and Dicarboxylate Metabolism

In a test performed comparing the tissues of patients with CRC without CRC using formalin-fixed paraffin-embedded tissues, it was discovered that glyoxylate and dicarboxylate metabolism showed increased levels in tumor-depleted differentially abundant metabolites [29]. Thus, increases in glyoxylate and dicarboxylate metabolism may be correlated with the presence of CRC for early detection.

#### 3.1.3. Glycine, Serine, and Threonine Metabolism

Glycine and serine together are responsible for the pre-synthesis of proteins, nucleic acids, and lipids, which all impact the growth of cancerous cells [30]. Amelio and colleagues state that the analysis of the pathway has revealed that its hyperactivation fuels the process of oncogenesis.

#### 3.1.4. Phenylalanine, Tyrosine, and Tryptophan Biosynthesis

**Figure 2:**
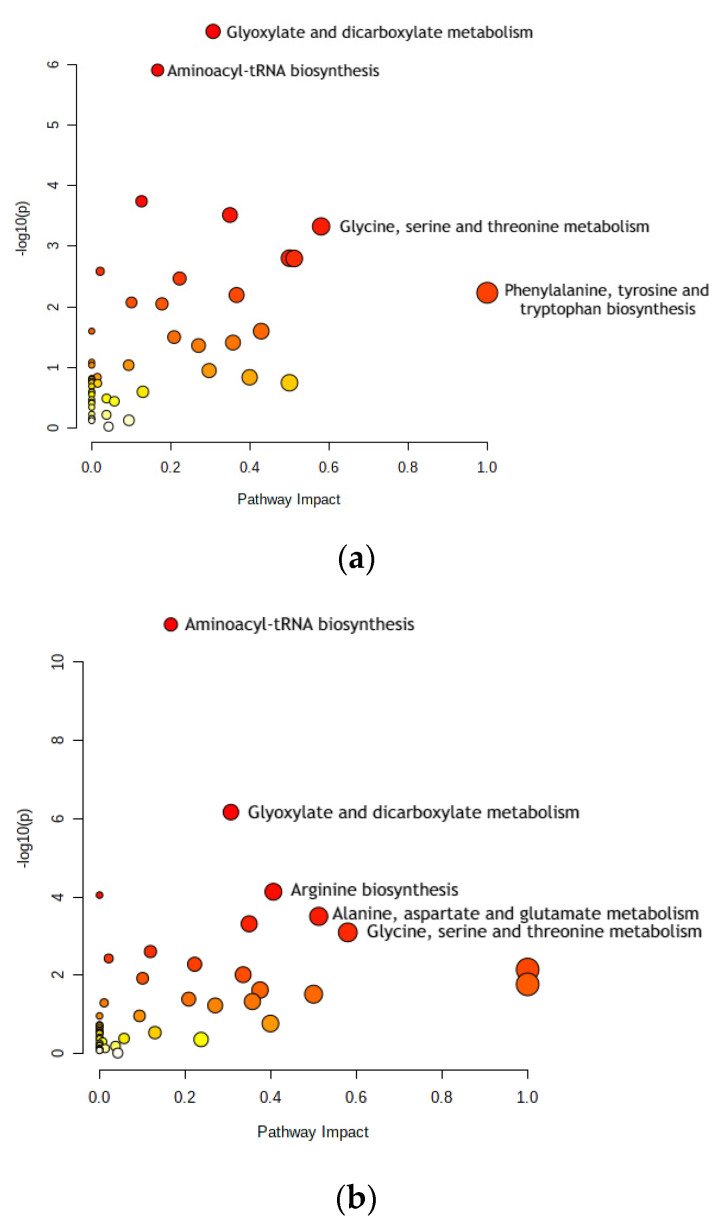
Dot plots for metabolic pathway analysis. Along the *X*-axis is pathway impact, and along the *Y*-axis is significance (*p*-value). The node size reflects significance, and the node color reflects pathway impact—the brighter the color, the greater is impact. The most significant pathways are labeled. (**a**) Stage 0–2 pathways: aminoacyl-tRNA biosynthesis; glyoxylate and dicarboxylate metabolism; glycine, serine, and threonine metabolism; and phenylalanine, tyrosine, and tryptophan biosynthesis. (**b**) Stage 3–4 pathways: aminoacyl-tRNA biosynthesis; glyoxylate and dicarboxylate metabolism; arginine biosynthesis; alanine, aspartate, and glutamate metabolism; and glycine, serine, and threonine metabolism. Pathway impact indicates how a series of actions among molecules in a cell might result in a change or specific product in a cell.

Phenylalanine, tyrosine, and tryptophan are present only in Stages 0–2 (see Figure 2a). They are aromatic amino acids (AAAs) that are involved in protein synthesis [31]. It was also reported that phenylalanine, tyrosine, and tryptophan (as metabolites) have been shown to have significantly different levels in patients with CRC and without it; this was also proven by many other studies [32].

#### 3.1.5. Arginine Biosynthesis

Arginine biosynthesis was present only in Stages 3–4 (see Figure 2b). The molecules of the arginine metabolic pathway are currently considered as targets for CRC treatments, such as chemoprevention or therapy. Reducing arginine through consumption and inhibiting the activity of one of the pathway’s main enzymes, ornithine decarboxylase (ODC), significantly decreases polyamine synthesis and thus the risk of CRC [33].

#### 3.1.6. Alanine, Aspartate, and Glutamate Metabolism

Alanine, aspartate, and glutamate metabolism is present only in Stages 3–4 (see Figure 2b). Comparing gastric and colon cancer tissues, levels of all free amino acids except for aspartate, glutamate, and glutamine were notably lower in gastric cancer [34]. In addition, tadalafil, a PDE5 inhibitor, was tested on human CRC cells to analyze its anti-tumor effect. It was concluded that alanine, aspartate, and glutamate metabolism may be the most significant factor in how tadalafil’s anti-tumor activity works, which has pharmaceutical potential for future cancer treatment [35].

#### 3.1.7. Differences in Metabolic Pathways for CRC Stages

While both Stages 0–2 and Stages 3–4 shared the pathways of aminoacyl-tRNA biosynthesis, glyoxylate and dicarboxylate metabolism, and glycine, serine, and threonine metabolism, the difference in these prominent pathways is the change from phenylalanine, tyrosine, and tryptophan biosynthesis to alanine, aspartate, and glutamate metabolism and arginine biosynthesis. This difference may arise from a change in key metabolite concentrations from the earlier to the later stages of CRC. Since the same random metabolite set was used for both groups CRC—Stages 0–1 and Stages 3–4—this could only mean that the differences in the selected metabolites for Stages 0–2 and Stages 3–4 are causing this shift. Stages 0–2’s unique metabolites are 3-hydroxy-butyrate, n-caprylic acid, acetylsalicylic acid, creatinine, ribulose, taurine, putrescine, 4-hydroxymandelate, *O*-phosphoethanolamine, gulcono-1,4-lactone, gallic acid, palmitoleate, and elaidic acid. Stages 3–4’s selected metabolites are glycolic acid, keto isoleucine 1, phosphate, leucine, proline, trans-4-hydroxy-L-proline, β-glutamic acid, xylose-2, lyxose-2, asparagine, 1,6-anhydroglucose, glycyl-glycine 1, citrulline, 1,5-anhydro-D-glucitol, lysine (4TMS), *N*-α-acetyl-L-ornithine 2, *N*-α-acetyl-L-lysine 2, tryptophan, and lactitol.

### 3.2. Machine-Learning Classification

**Figure 3:**
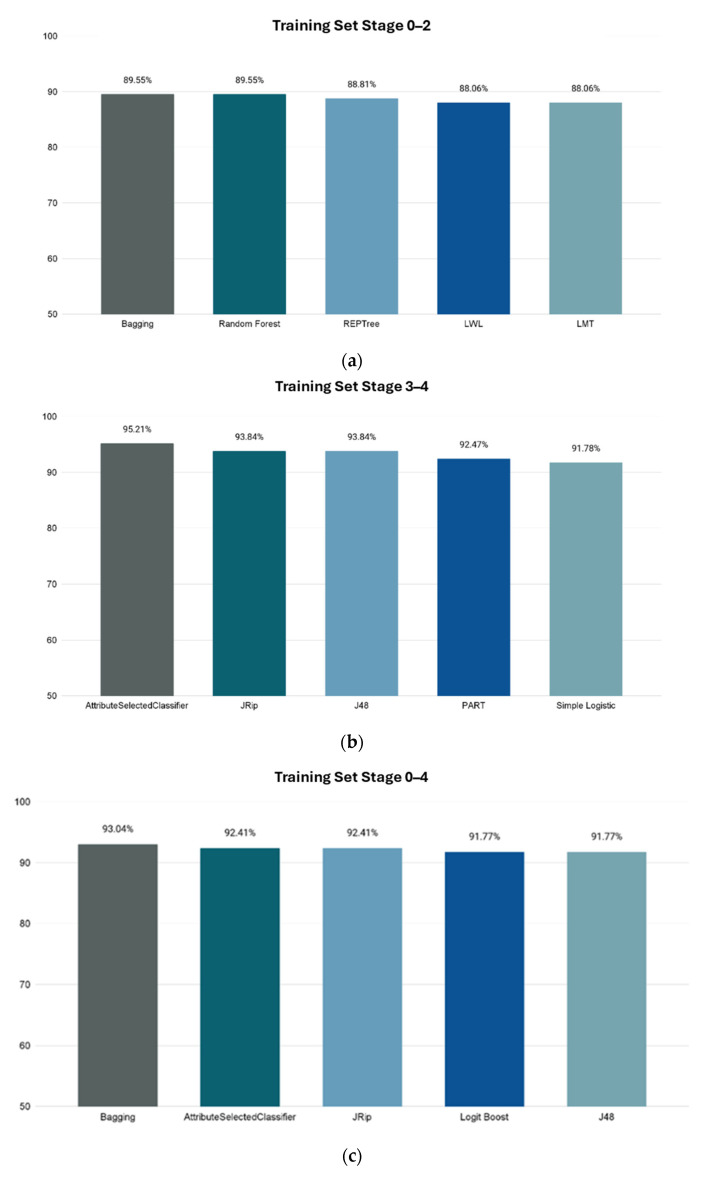
Accuracies of ML model’s prediction of metabolites’ correlation to CRC using 10-fold cross validation. *X*-axis shows the name of WEKA classification method, and *Y*-axis shows prediction accuracy percentage. (**a**) Stages 0–2. (**b**) Stages 3–4. (**c**) Stages 0–4.

Recently, the use of machine-learning techniques has become popular in the applications regarding biomedical purposes, including biomarker-based diagnostics, drug discovery, etc. For this study, the final dataset was composed of selected metabolites from publicly available data plus an equal number of random compounds. Using the InfoGainAttributeEval function, the original 3679 attributes were filtered down to 937 for all stages. Each of the filtered datasets for Stages 0–2, Stages 3–4, and Stages 0–4 was then tested with multiple classification algorithms available in the WEKA. The trained models were tested using 10-fold cross validation, with the accuracy of metabolite class prediction as the evaluation metric of choice. The best-performing models were as follows: Bagging classification for Stages 0–2 with 89.55% accuracy; AttributeSelectedClassifier classification for Stages 3–4 with 95.21% accuracy; and Bagging classification for Stages 0–4 with 93.04% accuracy. The results are shown in Figure 3. The receiver operating characteristic (ROC) and Precision–Recall curves and areas under them of the best classifiers are presented in Figures S1–S3 and S4–S6 accordingly.

On the next step, we conducted tests of our trained models on independent datasets. We used the best-performing trained models for each stage—Bagging classifier for Stages 0–2 and 0–4, and AttributeSelectedClassifier classifier for Stages 3–4—to evaluate unseen data.

The ML systems perform intrinsic cleaning of data, discarding noise information. Our system is trained to discriminate between the patients that have CRC and patients without it. Thyroid cancer is significantly different from CRC. This is why it was selected—to demonstrate that our ML program would clearly not select it as a diagnosis (having low accuracy of prediction) using the CRC trained system. So, it recognizes only CRC. The reliability of the system is demonstrated by its high accuracy of CRC diagnosis on a completely independent new dataset of metabolic biomarkers related to CRC. The clinical challenges may be met when the system is tested in clinical conditions.

The results are as follows.

**Figure 4:**
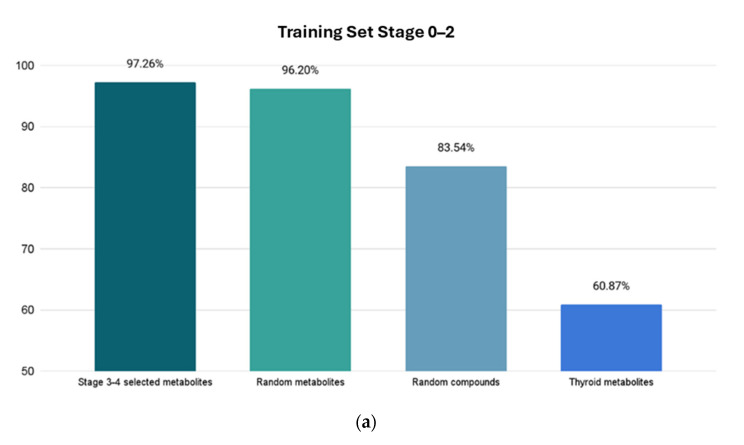

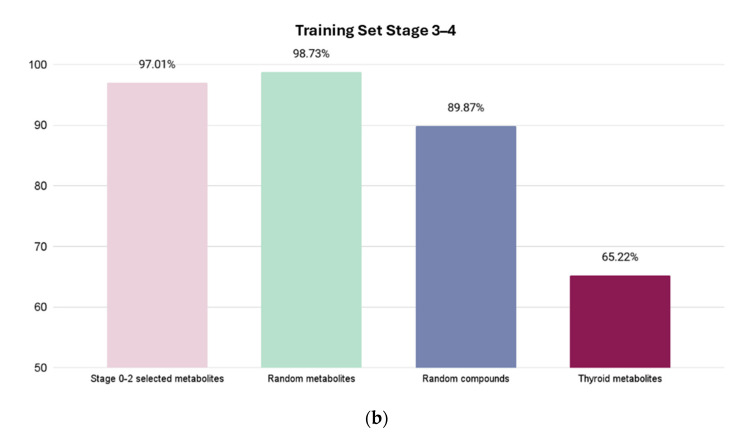
Resulting accuracies from four test sets. *X*-axis shows which test set is used, and *Y*-axis shows prediction accuracy percentage for highest accuracy models for Stages 0–2 and Stages 3–4. The first column shows percentage of correctly identified metabolites related to CRC. The next two columns show percentage of correctly identified metabolites as “random” metabolites. The last column shows the prediction score of the unrelated cancer metabolites with a much lower accuracy, demonstrating the high selectivity of the model. (**a**) Using trained Stage 0–2 Bagging classifier ML model. (**b**) Using trained Stage 3–4 AttributeSelectedClassifier ML model.

The Stage 0–2 Bagging classifier ML model was tested on 4 sets of data: (1) 146 Stage 3–4 metabolites; (2) 79 random HMDB metabolites; (3) 79 random ZINC compounds; and (4) a set of 23 thyroid cancer metabolites [33]. The resulting accuracies of metabolite class prediction are shown in Figure 4a.

For the Stage 3–4 model (trained with the AttributeSelectedClassifier), four sets of tests were conducted: (1) 134 Stage 0–2 metabolites; (2) 79 random HMDB metabolites; (3) 79 random ZINC compounds; and (4) 23 thyroid cancer metabolites. The Stage 0–2 metabolite test yielded a predictive accuracy of 97.01% (65 out of 67 metabolites correctly labeled as selected), the random metabolite test yielded a predictive accuracy of 98.73% (78 out of 79 metabolites correctly labeled as random), the random compound test yielded a predictive accuracy of 89.87% (71 out of 79 correctly labeled as random), and the thyroid metabolite test yielded a predictive accuracy of 65.22% (15 out of 23 metabolites labeled correctly as non-CRC).

### 3.3. Analysis of Protein Targets of miRNAs

We submitted the CRC Stage 3–4 metabolites, which were used for training the ML model for diagnostics of CRC, to the Drift program and obtained a list of 58 protein targets, which we selected using a threshold of >0.35.

**Figure 5:**
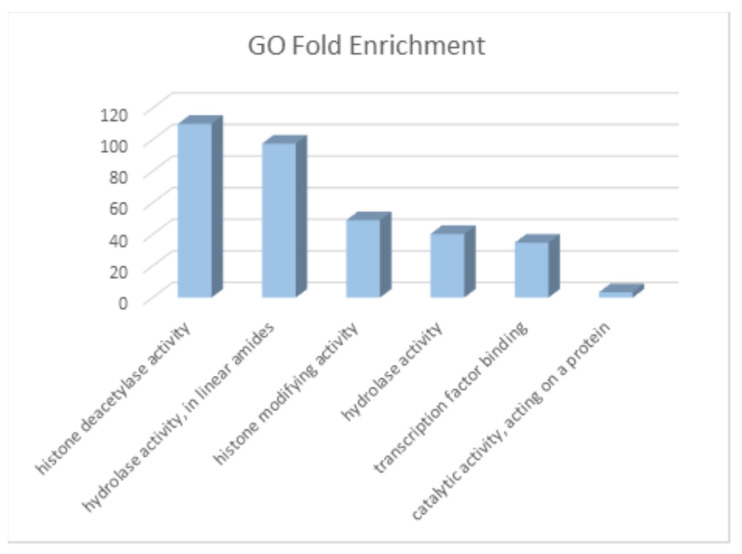
Fold enrichment of molecular functions of genes/targets of CRC metabolites. The greatest enrichments are related to epigenetic regulation.

Elucidated genes were analyzed with the Gene-Ontology (GO)-Panther Enrichment module. The results are presented in Figure 5. Note that the top enrichment scores have epigenetic regulation molecular functions. There are several publications that point out that metabolites can cause epigenetic regulation events that can be related to cancer [36,37].

**Figure 6:**
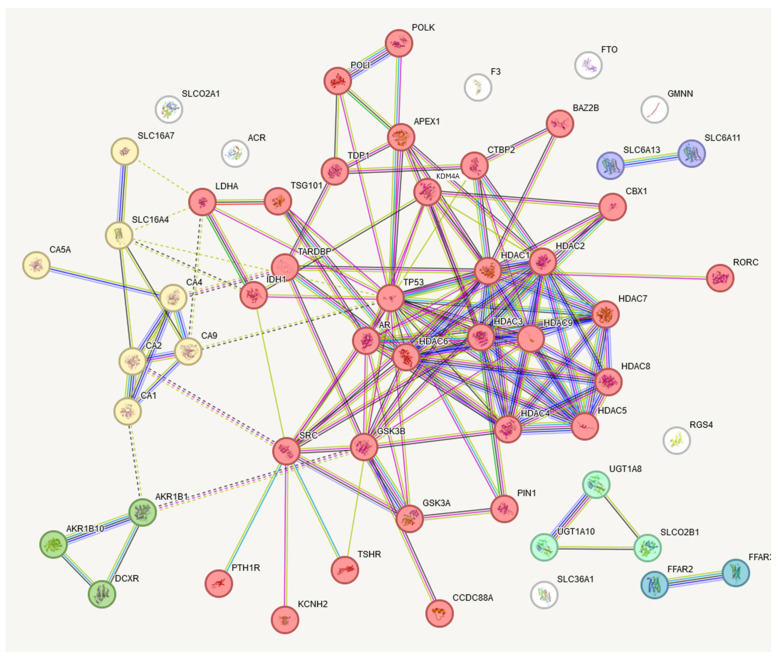
STRING presentation of gene targets of CRC metabolites. Six clusters were elucidated, with the largest having thirty-two interacting genes. This largest target contains many epigenetic regulation-related genes. The names of these genes are presented in Table 1.

The obtained set of protein targets was submitted to the STRING program, and we obtained a network of protein–protein interactions based on the predicted CRC metabolite gene targets (Figure 6). The obtained protein targets were also analyzed with the DisGeNET program to check their relation to various cancers. The results of this analysis show that 34 from 56 of the predicted gene targets have a known relation to different types of cancers, including CRC (Table 1). We did not expect that we would find only the neoplasms related to CRC, but, considering a lot of common genes involved in different cancers, we obtained a pattern corresponding to the current stage of research, where some of the cancers were explored more in depth than CRC. In general, these results support our assumption that the metabolites found in CRC comprise active agents affecting cancer-related genes. However, this finding needs further study.

We analyzed the set of genes selected from the CRC-related metabolites using the program STRING with the program PathBIX [27]. The resulting Table 2 presents the main pathways elucidated to be related to these metabolites.

This table shows that CRC-related metabolites are significantly related to the Notch, Wnt, and TGF-beta signaling pathways and can participate in several types of cancer. Interestingly, they participate in the transcriptional misregulation in cancers. These results open the way for a further elucidation of the roles of metabolites in the mechanisms of cancer, and at least support the concept of using them as cancer biomarkers.

## 4. Discussion

We developed a range of machine-learning models for the diagnostics of CRC, using sets of metabolites as biomarkers. As expected, the accuracies of the ML models trained on the CRC metabolites data from Stages 3–4 were greater than from Stages 0–2.

The accuracy for Stages 0–4 was also significant, but for the purpose of clinical use, it would be not too helpful because it composes the model for both the early and the late stages of cancer. It must be noted that the metabolites used for the elaboration of ML models participate in the pathways directly related to cancer development and are not just biomarkers. We elucidated several cancer-related pathways where these metabolites are directly involved. Testing trained models with a completely independent dataset of metabolites related to CRC showed significant recognition accuracy. Also, testing with random metabolites and metabolites related to the other cancer showed a much lower recognition accuracy of random metabolites from HMDB and a low recognition accuracy of other cancer types—such as thyroid cancer. This demonstrated the significant selectivity of the used models. We recommend this strategy for testing in medical practice.

In summary, the metabolite analysis of blood serum has proven to be a robust diagnostic method for colorectal cancer. Significant accuracy in machine-learning classifier models was shown for identifying CRC-correlated metabolites in the body; thus, this opens new avenues for further research to possibly develop novel treatment options or alternatives to the current, invasive methods that are used, such as colonoscopy. More studies and research will be able to expand the list of metabolites known to be connected to the presence of cancerous cells, thus establishing metabolite analysis as a propitious, accurate, and non-invasive screening method.

## Figures and Tables

**Table 1:** Clusters of gene targets of CRC metabolites.

Bubble	Cluster ID	Gene Count	Protein Names
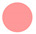	Cluster 1	32	APEX1, AR, BAZ2B, CBX1, CCDC88A, CTBP2, GSK3B, HDAC1, HDAC2, HDAC3, HDAC4, HDAC5, HDAC6, HDAC7, HDAC8, HDAC9, IDH1, KCNH2, KDM4A, LDHA, PIN1, POLI, POLK, PTH1R, RORC, SRC, TARDBP, TDP1, TP53, TGS101, TSHR
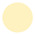	Cluster 2	7	CA1, CA2, CA4, CA5A, CA9, SLC16A4, SLC16A7
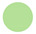	Cluster 3	3	AKR1B1, AKR1B10, DCXR
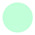	Cluster 4	3	SLCO2B1, UGT1A8, UGT1A10
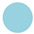	Cluster 5	2	FFAR2, FFAR3
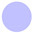	Cluster 6	2	SLC6A11, SLC6A13

**Table 2:** Signaling pathway related to the gene targets of CRC metabolites (Cluster 1).

Pathway	FWER	FDR
Notch Signaling Pathway	1.95 × 10^−8^	1.95 × 10^−8^
Wnt Signaling Pathway	1.76 × 10^−5^	8.29 × 10^−6^
TGF-Beta Signaling Pathway	2.49 × 10^−5^	8.29 × 10^−6^
Basal Cell Carcinoma	6.33 × 10^−5^	1.58 × 10^−5^
Influenza A	3.15 × 10^−3^	5.24 × 10^−4^
MicroRNAs in Cancer	3.72 × 10^−3^	5.31 × 10^−4^
Signaling Pathways Regulating Pluripotency of Stem Cells	5.51 × 10^−3^	6.89 × 10^−4^
Inflammatory Bowel Disease	9.86 × 10^−3^	1.10 × 10^−3^
Transcriptional Misregulation in Cancer	0.01	1.12 × 10^−3^
Gastric Cancer	0.01	1.27 × 10^−3^
Breast Cancer	0.02	1.50 × 10^−3^
Prostate Cancer	0.04	2.93 × 10^−3^

A *p*-value threshold of 0.05 yields an FDR of 5% among all truly null features. FDR, false discovery rate; FWER, family-wise error rate.

## Data Availability

All data from this study were sourced from public sources. Materials used in this study are available in the Supplementary Materials.

## Supplementary Materials

The following supporting information can be downloaded at: https://www.mdpi.com/article/10.3390/cimb46050254/s1. Figures S1–S3: Area under the Receiver Operating Characteristic Curve: Figure S1. Stage 0–2 after InfoGain filtration, Bagging Classifier; Figure S2. Stage 0–4 after InfoGain filtration, Bagging Classifier; Figure S3. Stage 0–2 after InfoGain filtration, AttributeSelectionClassifier; Figures S4–S6: Area under the Precision–Recall Curve: Figure S4. Stage 0–2 after InfoGain filtration, Bagging Classifier; Figure S5. Stage 0–4 after InfoGain filtration, Bagging Classifier; Figure S6. Stage 3–4 after InfoGain filtration, AttributeSelectionClassifier; Table S1. Stage 0–2 Metabolites; Table S2. Stage 3–4 Metabolites; Table S3. Stage 0–4 Metabolites; Table S4. Selected Machine-Learning Classifier Descriptions.

## Abbreviations

^1^H-NMR, proton nuclear magnetic resonance; AAA, aromatic amino acid; CRC, colorectal cancer; FDR, false discovery rate; FWER, family-wise error rate; GO, gene ontology; KEGG, Kyoto Encyclopedia of Genes and Genomes; LMT, logistic model tree; LWL, locally weighted learning; ML, machine learning; MSA, multivariate statistical analysis; ODC, ornithine decarboxylase; PDE5, phosphodiesterase-5; ROC, receiver operating characteristic; SMILES, simplified molecular-input line-entry system; VIP, variable importance of projection.
