# Supplementary material for: Colorectal Cancer Detection via Metabolites and Machine Learning
Source: Curr Issues Mol Biol. 2024 Apr 30;46(5):4133–46. doi: 10.3390/cimb46050254 (PMC11119033; doi:10.3390/cimb46050254)
Supplement: Supplementary file 1 [file cimb-46-00254-s001.zip › cimb-2952042-supplementary.pdf]

Colorectal Cancer Detection via Metabolites and Machine Learning

Rachel Yang , Igor F. Tsigelny, Santosh Kesari, Valentina L. Kouznetsova

Supplementary Material 1

Figures S1–S6

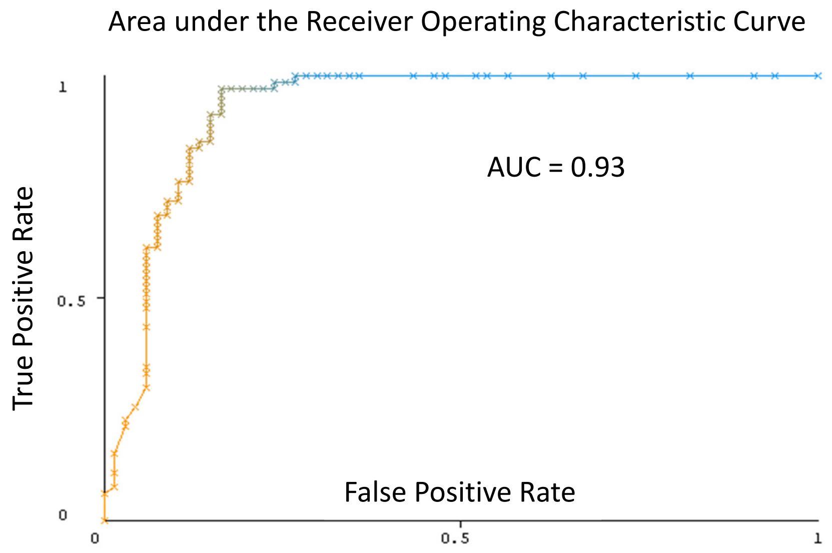

Figure S1. Stage 0–2 after InfoGain filtration, Bagging Classifier

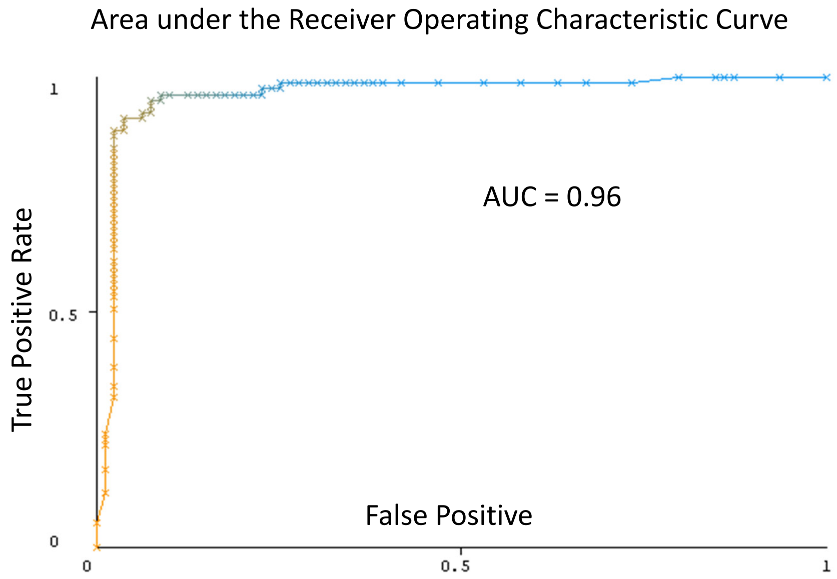

Figure S2. Stage 0–4 after InfoGain filtration,

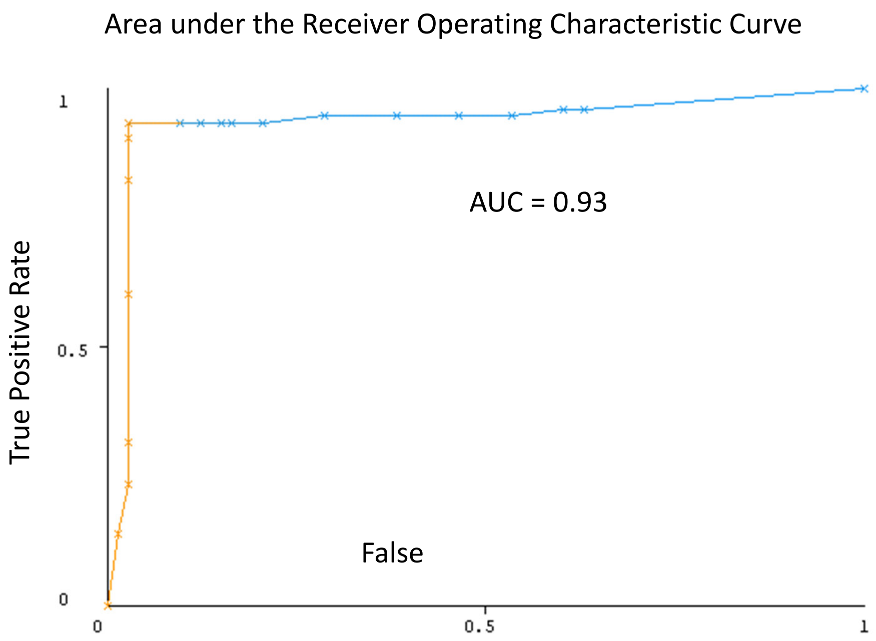

Figure S3. Stage 0–2 after InfoGain filtration,

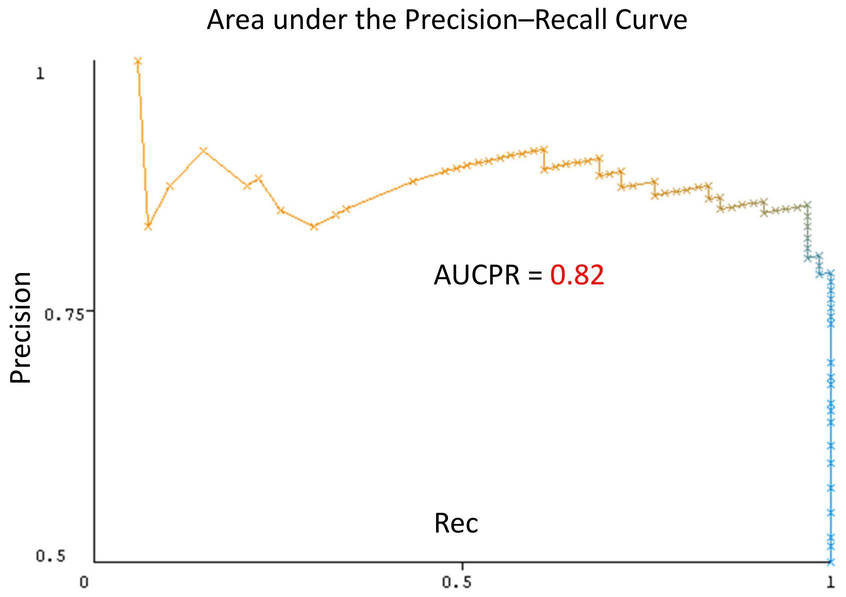

Figure S4. Stage 0–2 after InfoGain filtration,

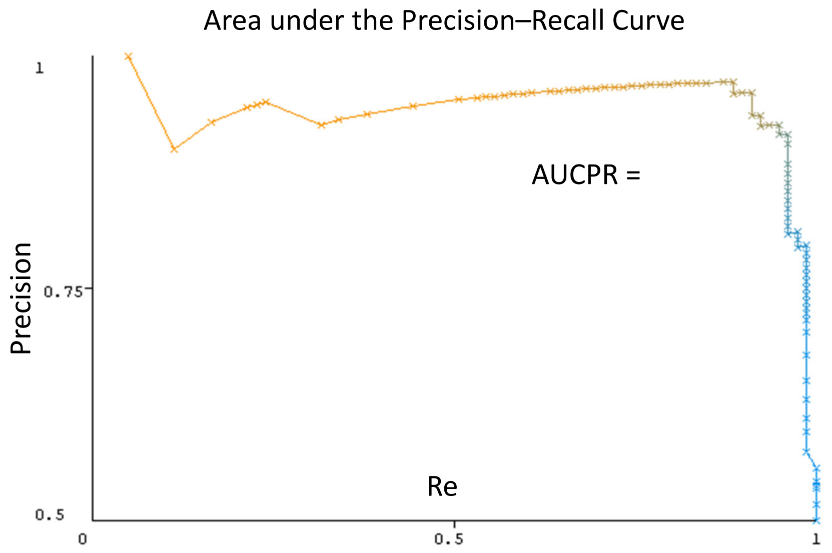

Figure S5. Stage 0–4 after InfoGain filtration,

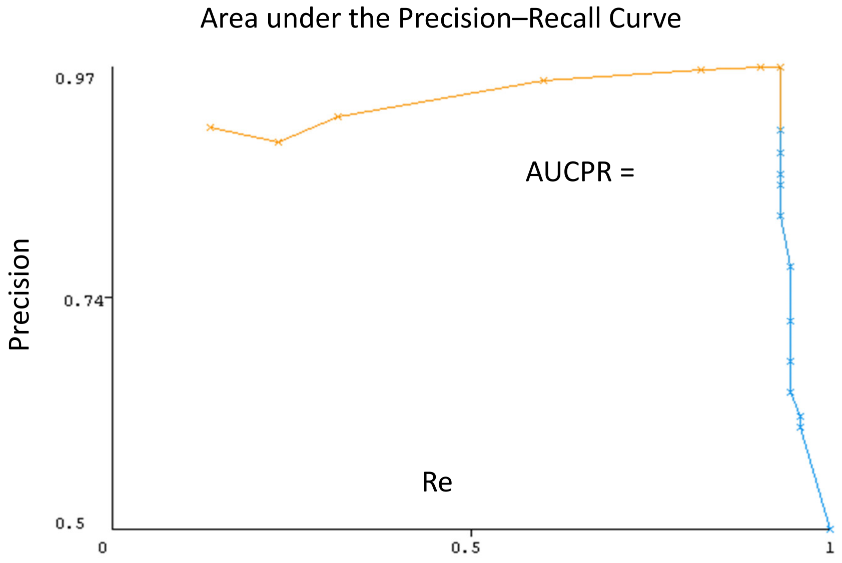

Figure S6. Stage 3–4 after InfoGain filtration,

Colorectal Cancer Detection via Metabolites and Machine Learning

Rachel Yang , Igor F. Tsigelny, Santosh Kesari, Valentina L. Kouznetsova

Supplementary Material 2

Tables S1–S4

Table S1. Stage 0–2 Metabolites

#	Name	Fold Change	SMILES (canonical)
1	Pyruvate	1.53	CC(=O)C(=O)[O−]
2	Oxalacetic acid	1.53	C(C(=O)C(=O)O)C(=O)O
3	Lactic acid	0.74	CC(C(=O)O)O
4	2-hydroxy-butyrate	1.35	CCC(C(=O)[O−])O
5	Oxalate	1.91	C(=O)(C(=O)[O−])[O−]
6	Sarcosine	1.41	CNCC(=O)O
7	3-hydroxy-butyrate	1.54	CC(CC(=O)[O−])O
8	Valine(2TMS)	1.09	CC(C)C(C(=O)O[Si](C)(C)C)N[Si](C)(C)C
9	Dihydroxyacetone	1.09	C(C(=O)CO)O
10	2-aminoethanol	1.12	C(CO)N
11	n-caprylic acid	1.17	CCCCCCCC(=O)O
12	Isoleucine	1.26	CCC(C)C(C(=O)O)N
13	Glycine(3TMS)	1.16	C[Si](C)(C)N(CC(=O)O[Si](C)(C)C)[Si](C)(C)C
14	Glyceric acid	1.31	C(C(C(=O)O)O)O
15	Serine(3TMS)	1.32	C[Si](C)(C)NC(CO[Si](C)(C)C)C(=O)O[Si](C)(C)C
16	Nonanoic acid(C9)	0.79	CCCCCCCCC(=O)O
17	ß-Alanine	1.33	C(CN)C(=O)O
18	Malic acid	1.28	C(C(C(=O)O)O)C(=O)O
19	Threitol	1.38	C(C(C(CO)O)O)O

20	meso-erythritol	2.03	C(C(C(CO)O)O)O
21	Acetylsalicylic acid	0.64	CC(=O)OC1=CC=CC=C1C(=O)O
22	Aspartic acid	1.47	C(C(C(=O)O)N)C(=O)O
23	Pyroglutamic acid	1.44	C1CC(=O)NC1C(=O)O
24	Creatinine	0.85	CN1CC(=O)N=C1N
25	Glutamic acid	1.57	C(CC(=O)O)C(C(=O)O)N
26	Phenylalanine	1.26	C1=CC=C(C=C1)CC(C(=O)O)N
27	p-hydroxybenzoic acid	1.65	C1=CC(=CC=C1C(=O)O)O
28	Threo-β-hydroxyaspartic acid	1.45	C(C(C(=O)O)O)(C(=O)O)N
29	Arabinose	1.48	C1C(C(C(C(O1)O)O)O)O
30	Lauric acid	1.33	CCCCCCCCCCCC(=O)O
31	Ribulose	0.66	C(C(C(C(=O)CO)O)O)O
32	Ribose	0.66	C(C(C(C(C=O)O)O)O)O
33	Taurine	4.63	C(CS(=O)(=O)O)N
34	Xylitol	1.28	C(C(C(C(CO)O)O)O)O
35	Arabitol	1.31	C(C(C(C(CO)O)O)O)O
36	Ribitol	1.35	C(C(C(C(CO)O)O)O)O
37	Putrescine	0.72	C(CCN)CN
38	Aconitate	1.20	C(C(=CC(=O)O)C(=O)O)C(=O)O
39	4-hydroxymandelate	0.91	C1=CC(=CC=C1C(C(=O)O)O)O
40	Methoxy-4-hydroxyphenylacetate	0.80	COC1=C(C=CC(=C1)CC(=O)O)O
41	O-phosphoethanolamine	0.92	C(COP(=O)(O)O)N
42	Citric acid	1.28	C(C(=O)O)C(CC(=O)O)(C(=O)O)O
43	Isocitric acid	1.28	C(C(C(C(=O)O)O)C(=O)O)C(=O)O
44	Ornithine	1.36	C(CC(C(=O)O)N)CN

45	Tagatose_2(or Psicose_2)	1.57	C(C(C(C(C(=O)CO)O)O)O)O
46	α-sorbopyranose_1 (or Fructose_1)	1.13	C1C(C(C(C(O1)(CO)O)O)O)O
47	Mannose_1	1.26	C(C(C(C(C(C=O)O)O)O)O)O
48	5-dehydroquinic acid	1.23	C1C(C(C(=O)CC1(C(=O)O)O)O)O
49	Glucose_1	0.73	C(C1C(C(C(C(O1)O)O)O)O)O
50	Gulcono-1,4-lactone	1.88	C(C(C1C(C(C(=O)O1)O)O)O)O
51	Galactosamine_1	1.59	CC(=O)NC1C(C(C(OC1O)CO)O)O
52	Glucuronate_1	1.34	C(=O)C(C(C(C(C(=O)O)O)O)O)O
53	Glucosamine_2	1.28	C(C1C(C(C(C(O1)O)N)O)O)O
54	Tyrosine	1.27	C1=CC(=CC=C1CC(C(=O)O)N)O
55	Gallic acid	0.81	C1=C(C=C(C(=C1O)O)O)C(=O)O
56	1-hexadecanol	1.15	CCCCCCCCCCCCCCCCO
57	S-benzyl-L-Cysteine_1	1.02	C1=CC=C(C=C1)CSCC(C(=O)O)N
58	Palmitoleate	1.44	CCCCCCC=CCCCCCCCC(=O)[O−]
59	Dopamine	1.96	C1=CC(=C(C=C1CCN)O)O
60	Inositol	1.20	C1(C(C(C(C(C1O)O)O)O)O)O
61	Heptadecanoate	1.30	CCCCCCCCCCCCCCCCC(=O)[O−]
62	Kynurenine	1.72	C1=CC=C(C(=C1)C(=O)CC(C(=O)O)N)N
63	Cystamine	1.43	C(CSSCCN)N
64	Elaidic acid	1.13	CCCCCCCCC=CCCCCCCCC(=O)O
65	Cysteine	1.72	C(C(C(=O)O)N)S
66	Cystine	1.72	C(C(C(=O)O)N)SSCC(C(=O)O)N
67	2'-deoxyuridine_2	1.37	C1C(C(OC1N2C=CC(=O)NC2=O)CO)O

REFERENCE: Table compiled from Nishiumi, S.; Kobayashi, T.; Ikeda, A.; Yoshie, T.; Kibi, M.; Izumi, Y.; Okuno, T.; Hayashi, N.; Kawano, S.; Takenawa, T.;et al. A novel serum metabolomics-based diagnostic approach for colorectal cancer. *PLoS ONE*. **2012**, *7*, **e40459**. https://doi.org/10.1371/journal.pone.0040459.

Table S2. Stage 3–4 Metabolites

#	Name	Fold Change	SMILES (canonical)
1	Pyruvate	1.53	CC(=O)C(=O)[O−]
2	Oxalacetic acid	1.53	C(C(=O)C(=O)O)C(=O)O
3	Lactic acid	0.83	CC(C(=O)O)O
4	Glycolic acid	1.22	C(C(=O)O)O
5	2-hydroxy-butyrate	1.51	CCC(C(=O)[O−])O
6	Oxalate	1.45	C(=O)(C(=O)[O−])[O−]
7	Sarcosine	1.69	CNCC(=O)O
8	Ketoisoleucine_1	1.25	CCC(C)C(=O)C(=O)O
9	Valine(2TMS)	1.14	CC(C)C(C(=O)O[Si](C)(C)C)N[Si](C)(C)C
10	Dihydroxyacetone	1.39	C(C(=O)CO)O
11	2-aminoethanol	1.15	C(CO)N
12	Phosphate	1.27	[O−]P(=O)([O−])[O−]
13	Leucine	1.23	CC(C)CC(C(=O)O)N
14	Isoleucine	1.46	CCC(C)C(C(=O)O)N
15	Proline	1.33	C1CC(NC1)C(=O)O
16	Glycine(3TMS)	1.15	C[Si](C)(C)N(CC(=O)O[Si](C)(C)C)[Si](C)(C)C
17	Glyceric acid	1.29	C(C(C(=O)O)O)O
18	Serine(3TMS)	1.42	C[Si](C)(C)NC(CO[Si](C)(C)C)C(=O)O[Si](C)(C)C
19	Nonanoic acid(C9)	0.77	CCCCCCCCC(=O)O
20	ß-Alanine	1.26	C(CN)C(=O)O
21	Malic acid	1.47	C(C(C(=O)O)O)C(=O)O
22	Threitol	1.32	C(C(C(CO)O)O)O
23	meso-erythritol	1.99	C(C(C(CO)O)O)O
24	Aspartic acid	1.93	C(C(C(=O)O)N)C(=O)O
25	trans-4-hydroxy-L-proline	1.32	C1C(CNC1C(=O)O)O
26	Pyroglutamic acid	1.25	C1CC(=O)NC1C(=O)O
27	β-Glutamic acid	1.19	C(C(CC(=O)O)N)C(=O)O
28	Glutamic acid	2.38	C(CC(=O)O)C(C(=O)O)N

29	Phenylalanine	1.59	C1=CC=C(C=C1)CC(C(=O)O)N
30	p-hydroxybenzoic acid	1.53	C1=CC(=CC=C1C(=O)O)O
31	Xylose_2	1.15	C(C(C(C(C=O)O)O)O)O
32	Lyxose_2	1.52	C(C(C(C(C=O)O)O)O)O
33	Threo-β-hydroxyaspartic acid	1.55	C(C(C(=O)O)O)(C(=O)O)N
34	Arabinose	1.56	C1C(C(C(C(O1)O)O)O)O
35	Lauric acid	1.15	CCCCCCCCCCCC(=O)O
36	Ribose	0.76	C(C(C(C(C=O)O)O)O)O
37	Asparagine	1.23	C(C(C(=O)O)N)C(=O)N
38	Xylitol	1.46	C(C(C(C(CO)O)O)O)O
39	1,6-anhydroglucose	0.96	C1C2C(C(C(C(O1)O2)O)O)O
40	Arabitol	1.39	C(C(C(C(CO)O)O)O)O
41	Ribitol	1.49	C(C(C(C(CO)O)O)O)O
42	Aconitate	1.21	C(C(=CC(=O)O)C(=O)O)C(=O)O
43	Methoxy-4-hydroxyphenylacetate	0.99	COC1=C(C=CC(=C1)CC(=O)O)O
44	Glycyl-Glycine_1	1.22	C(C(=O)NCC(=O)O)N
45	Citric acid	1.28	C(C(=O)O)C(CC(=O)O)(C(=O)O)O
46	Isocitric acid	1.28	C(C(C(C(=O)O)O)C(=O)O)C(=O)O
47	Ornithine	1.37	C(CC(C(=O)O)N)CN
48	Citrulline	1.29	C(CC(C(=O)O)N)CNC(=O)N
49	1,5-anhydro-D-glucitol	0.67	C1C(C(C(C(O1)CO)O)O)O
50	Tagatose_2(or Psicose_2)	1.82	C(C(C(C(C(=O)CO)O)O)O)O
51	α-sorbopyranose_1 (or Fructose_1)	2.10	C1C(C(C(C(O1)(CO)O)O)O)O
52	Mannose_1	1.83	C(C(C(C(C(C=O)O)O)O)O)O
53	5-dehydroquinic acid	1.77	C1C(C(C(=O)CC1(C(=O)O)O)O)O
54	Glucose_1	0.93	C(C1C(C(C(C(O1)O)O)O)O)O

55	Lysine(4TMS)	0.89	C[Si](C)(C)NC(CCCCN([Si](C)(C)C)[Si](C)(C)C)C(=O)O[Si](C)(C)C
56	Galactosamine_1	1.54	CC(=O)NC1C(C(C(OC1O)CO)O)O
57	Glucuronate_1	1.34	C(=O)C(C(C(C(C(=O)O)O)O)O)O
58	Glucosamine_2	1.28	C(C1C(C(C(C(O1)O)N)O)O)O
59	Tyrosine	1.22	C1=CC(=CC=C1CC(C(=O)O)N)O
60	1-hexadecanol	1.21	CCCCCCCCCCCCCCCCO
61	N-α-acetyl-L-Ornithine_2	1.41	CC(=O)NC(CCN)CC(=O)O
62	S-benzyl-L-Cysteine_1	1.41	C1=CC=C(C=C1)CSCC(C(=O)O)N
63	Dopamine	1.99	C1=CC(=C(C=C1CCN)O)O
64	Inositol	1.17	C1(C(C(C(C(C1O)O)O)O)O)O
65	N-α-acetyl-L-Lysine_2	2.08	CC(=O)NC(CCCCN)C(=O)O
66	Heptadecanoate	1.37	CCCCCCCCCCCCCCCCC(=O)[O−]
67	Kynurenine	1.98	C1=CC=C(C(=C1)C(=O)CC(C(=O)O)N)N
68	Cystamine	1.24	C(CSSCCN)N
69	Tryptophan	0.97	C1=CC=C2C(=C1)C(=CN2)CC(C(=O)O)N
70	Cysteine	1.72	C(C(C(=O)O)N)S
71	Cystine	1.72	C(C(C(=O)O)N)SSCC(C(=O)O)N
72	2'-deoxyuridine_2	1.71	C1C(C(OC1N2C=CC(=O)NC2=O)CO)O
73	Lactitol	8.19	C(C1C(C(C(C(O1)OC(C(CO)O)C(C(CO)O)O)O)O)O)O

REFERENCE: Table compiled from Nishiumi, S.; Kobayashi, T.; Ikeda, A.; Yoshie, T.; Kibi, M.; Izumi, Y.; Okuno, T.; Hayashi, N.; Kawano, S.; Takenawa, T.;et al. A novel serum metabolomics-based diagnostic approach for colorectal cancer. *PLoS ONE*. **2012**, *7*, **e40459**. https://doi.org/10.1371/journal.pone.0040459.

Table S3. Stage 0–4 Metabolites

#	**Name**	**Fold Change**	**SMILES (canonical)**
1	Pyruvate	1.53	CC(=O)C(=O)[O−]
2	Oxalacetic acid	1.53	C(C(=O)C(=O)O)C(=O)O
3	Lactic acid	0.78	CC(C(=O)O)O
4	Glycolic acid	1.13	C(C(=O)O)O

5	2-hydroxy-butyrate	1.42	CCC(C(=O)[O−])O
6	Oxalate	1.70	C(=O)(C(=O)[O−])[O−]
7	Sarcosine	1.54	CNCC(=O)O
8	3-hydroxy-butyrate	1.88	CC(CC(=O)[O−])O
9	Ketoisoleucine_1	1.17	CCC(C)C(=O)C(=O)O
10	Valine(2TMS)	1.11	CC(C)C(C(=O)O[Si](C)(C)C)N[Si](C)(C)C
11	Dihydroxyacetone	1.23	C(C(=O)CO)O
12	2-aminoethanol	1.13	C(CO)N
13	n-caprylic acid	1.13	CCCCCCCC(=O)O
14	Phosphate	1.16	[O−]P(=O)([O−])[O−]
15	Leucine	1.16	CC(C)CC(C(=O)O)N
16	Isoleucine	1.35	CCC(C)C(C(=O)O)N
17	Proline	1.23	C1CC(NC1)C(=O)O
18	Glycine(3TMS)	1.16	C[Si](C)(C)N(CC(=O)O[Si](C)(C)C)[Si](C)(C)C
19	Glyceric acid	1.30	C(C(C(=O)O)O)O
20	Serine(3TMS)	1.36	C[Si](C)(C)NC(CO[Si](C)(C)C)C(=O)O[Si](C)(C)C
21	Nonanoic acid(C9)	0.78	CCCCCCCCC(=O)O
22	ß-Alanine	1.30	C(CN)C(=O)O
23	Malic acid	1.37	C(C(C(=O)O)O)C(=O)O
24	Threitol	1.35	C(C(C(CO)O)O)O
25	meso-erythritol	2.01	C(C(C(CO)O)O)O
26	Aspartic acid	1.68	C(C(C(=O)O)N)C(=O)O
27	trans-4-hydroxy-L-proline	1.15	C1C(CNC1C(=O)O)O
28	Pyroglutamic acid	1.35	C1CC(=O)NC1C(=O)O
29	Creatinine	0.90	CN1CC(=O)N=C1N
30	ß-Glutamic acid	1.13	C(C(CC(=O)O)N)C(=O)O
31	Glutamic acid	1.94	C(CC(=O)O)C(C(=O)O)N
32	Phenylalanine	1.41	C1=CC=C(C=C1)CC(C(=O)O)N
33	p-hydroxybenzoic acid	1.60	C1=CC(=CC=C1C(=O)O)O
34	Xylose_2	1.33	C(C(C(C(C=O)O)O)O)O

35	Threo-β-hydroxyaspartic acid	1.50	C(C(C(=O)O)O)(C(=O)O)N
36	Arabinose	1.52	C1C(C(C(C(O1)O)O)O)O
37	Lauric acid	1.25	CCCCCCCCCCCC(=O)O
38	Ribulose	0.71	C(C(C(C(=O)CO)O)O)O
39	Ribose	0.71	C(C(C(C(C=O)O)O)O)O
40	Asparagine	1.17	C(C(C(=O)O)N)C(=O)N
41	Taurine	3.43	C(CS(=O)(=O)O)N
42	Xylitol	1.36	C(C(C(C(CO)O)O)O)O
43	Arabitol	1.35	C(C(C(C(CO)O)O)O)O
44	Ribitol	1.42	C(C(C(C(CO)O)O)O)O
45	Putrescine	0.87	C(CCN)CN
46	Aconitate	1.20	C(C(=CC(=O)O)C(=O)O)C(=O)O
47	Methoxy-4-hydroxyphenylacetate	0.89	COC1=C(C=CC(=C1)CC(=O)O)O
48	O-phosphoethanolamine	0.95	C(COP(=O)(O)O)N
49	Citric acid	1.28	C(C(=O)O)C(CC(=O)O)(C(=O)O)O
50	Isocitric acid	1.28	C(C(C(C(=O)O)O)C(=O)O)C(=O)O
51	Ornithine	1.37	C(CC(C(=O)O)N)CN
52	Citrulline	1.18	C(CC(C(=O)O)N)CNC(=O)N
53	1,5-anhydro-D-glucitol	0.87	C1C(C(C(C(O1)CO)O)O)O
54	Tagatose_2(or Psicose_2)	1.68	C(C(C(C(C(=O)CO)O)O)O)O
55	α-sorbopyranose_1 (or Fructose_1)	1.57	C1C(C(C(C(O1)(CO)O)O)O)O
56	Mannose_1	1.52	C(C(C(C(C(C=O)O)O)O)O)O
57	5-dehydroquinic acid	1.47	C1C(C(C(=O)CC1(C(=O)O)O)O)O
58	Glucose_1	0.82	C(C1C(C(C(C(O1)O)O)O)O)O
59	Gulcono-1,4-lactone	2.04	C(C(C1C(C(C(=O)O1)O)O)O)O
60	Galactosamine_1	1.57	CC(=O)NC1C(C(C(OC1O)CO)O)O

61	Glucuronate_1	1.34	C(=O)C(C(C(C(C(=O)O)O)O)O)O
62	Glucosamine_2	1.28	C(C1C(C(C(C(O1)O)N)O)O)O
63	Tyrosine	1.25	C1=CC(=CC=C1CC(C(=O)O)N)O
64	Gallic acid	0.90	C1=C(C=C(C(=C1O)O)O)C(=O)O
65	1-hexadecanol	1.18	CCCCCCCCCCCCCCCCO
66	N-α-acetyl-L-Ornithine_2	1.30	CC(=O)NC(CCN)CC(=O)O
67	S-benzyl-L-Cysteine_1	1.19	C1=CC=C(C=C1)CSCC(C(=O)O)N
68	Palmitoleate	1.35	CCCCCCC=CCCCCCCCC(=O)[O−]
69	Dopamine	1.97	C1=CC(=C(C=C1CCN)O)O
70	Inositol	1.19	C1(C(C(C(C(C1O)O)O)O)O)O
71	N-α-acetyl-L-Lysine_2	1.68	CC(=O)NC(CCCCN)C(=O)O
72	Heptadecanoate	1.33	CCCCCCCCCCCCCCCCC(=O)[O−]
73	Kynurenine	1.83	C1=CC=C(C(=C1)C(=O)CC(C(=O)O)N)N
74	Cystamine	1.34	C(CSSCCN)N
75	Elaidic acid	1.14	CCCCCCCCC=CCCCCCCCC(=O)O
76	Cysteine	1.72	C(C(C(=O)O)N)S
77	Cystine	1.72	C(C(C(=O)O)N)SSCC(C(=O)O)N
78	2'-deoxyuridine_2	1.52	C1C(C(OC1N2C=CC(=O)NC2=O)CO)O
79	Lactitol	4.35	C(C1C(C(C(C(O1)OC(C(CO)O)C(C(CO)O)O)O)O)O)O

REFERENCE: Table compiled from Nishiumi, S.; Kobayashi, T.; Ikeda, A.; Yoshie, T.; Kibi, M.; Izumi, Y.; Okuno, T.; Hayashi, N.; Kawano, S.; Takenawa, T.;et al. A novel serum metabolomics-based diagnostic approach for colorectal cancer. *PLoS ONE*. **2012**, *7*, **e40459**. https://doi.org/10.1371/journal.pone.0040459

Table S4. Selected Machine-Learning Classifier Descriptions

**Bagging** – an ensemble learning method that divides training dataset into subsets and selects with replacement; used to reduced variance.
**Random Forest –** an ensemble learning method using multiple decision trees to reduce overfitting and variance.
**REPTree** (Reduced Error Pruning Tree) **–** a decision tree learning method to split and diminish error in criterion.
**LWL** (Locally Weighted Learning) **–** an algorithm that assigns instance weights to perform classification or regression.
**LMT** (Logistic Model Trees) **–** an algorithm that uses classification trees along with logistic regression functions at its leaves.
**AttributeSelectedClassifier –** a classification algorithm that identifies a best-splitting criterion to separate the data in the training set into separate classes to evaluate.
**JRip –** an incremental reduced-error pruning algorithm; uses rule-based classification to classify elements of the training set.
**J48 –** a classification algorithm using decision trees to choose the best attribute to partition the data training set.
**PART** (Partial C4.5) **–** a rule-based classifier that uses a decision tree to create rules from leaves and iterate through to evaluate given data.
**Simple Logistic –** a regression algorithm that predicts one binary variable based on one other variable to produce a numerical evaluation.
**Logit Boost –** an algorithm that performs additive logistic regression to evaluate data with multiple classes.
